# Building an IoT Platform Based on Service Containerisation

**DOI:** 10.3390/s21196688

**Published:** 2021-10-08

**Authors:** Mário Antunes, Ana Rita Santiago, Sérgio Manso, Diogo Regateiro, João Paulo Barraca, Diogo Gomes, Rui L. Aguiar

**Affiliations:** 1 Instituto de Telecomunicações, Departamento de Electrónica, Telecomunicações e Informática, Universidade de Aveiro, 3810-193 Aveiro, Portugal; diogoregateiro@av.it.pt (D.R.); jpbarraca@av.it.pt (J.P.B.); dgomes@av.it.pt (D.G.); ruilaa@av.it.pt (R.L.A.); 2 Departamento de Electrónica, Telecomunicações e Informática, Universidade de Aveiro, 3810-193 Aveiro, Portugal; ana.rita.santiago@ua.pt (A.R.S.); sergio.manso@ua.pt (S.M.)

**Keywords:** platform virtualisation, Internet of Things, ambient intelligence

## Abstract

IoT platforms have become quite complex from a technical viewpoint, becoming the cornerstone for information sharing, storing, and indexing given the unprecedented scale of smart services being available by massive deployments of a large set of data-enabled devices. These platforms rely on structured formats that exploit standard technologies to deal with the gathered data, thus creating the need for carefully designed customised systems that can handle thousands of heterogeneous data sensors/actuators, multiple processing frameworks, and storage solutions. We present the SCoT2.0 platform, a generic-purpose IoT Platform that can acquire, process, and visualise data using methods adequate for both real-time processing and long-term Machine Learning (ML)-based analysis. Our goal is to develop a large-scale system that can be applied to multiple real-world scenarios and is potentially deployable on private clouds for multiple verticals. Our approach relies on extensive service containerisation, and we present the different design choices, technical challenges, and solutions found while building our own IoT platform. We validate this platform supporting two very distinct IoT projects (750 physical devices), and we analyse scaling issues within the platform components.

## 1. Introduction

The Internet of Things (IoT) [1] is the general term applied to all networks of small sensing devices that are capable of sharing data and collaborating to achieve a common goal. It is built under a paradigm of integrating a variety of objects into a communication network (eventually across the Internet), bringing what once were proprietary solutions and protocols into a much more open and standard-compliant architecture. The differentiating factor between proprietary sensing solutions and the IoT concept is the potential of the latter to integrate an ever-increasing set of devices and/or services. One key player that benefits greatly from this paradigm is the industrial sector, which is being remodelled into a technological and data-centric world called *Industry 4.0* [2]. The underlying complexity of the industrial sector demands high-performance platforms capable of handling a high number of simultaneous connections, storing and processing the generated data.

Previously, we developed an IoT platform named Smart Cloud of Things (SCoT)v1 [3,4,5]. This was developed in close collaboration with Telecom providers, providing a service infrastructure implemented in a telecommunication framework. The platform provided a rich service execution environment that was built upon ETSI standards. This results in the facilitated orchestration of services and devices and integrated (graphical) portals for end customers.

As SCoTv1 was used in the deployment of IoT scenarios, it became evident that the European Telecommunications Standards Institute (ETSI) IoT standards were too complex and limited for the realisation of larger scenarios, which became commonplace in the intervening years. This led to several evolutions in our platform [5,6]. The first evolution was replacing the complex ETSI IoT standards with the agile Eclipse IoT. The second evolution was the addition of a modular data gateway service that allows persistent storage into multiple different storage solutions.

Nevertheless, these evolutions retained severe drawbacks from the initial design: lack of flexibility and vertical and horizontal scaling. This paper presents the latest iteration of the SCoT platform. The previous iterations were monolithic, which limited the scalability of the platform, an issue that became self-evident when the SCoTv1 was (tentatively) used for the deployment of two large IoT scenarios: *Smart Green Homes* (http://www.ua.pt/smartgreenhomes/ accessed on 1 October 2021) and *PASMO* (https://www.it.pt/Projects/Index/4508 accessed on 1 October 2021). *Smart Green Homes* aims to create IoT platforms and services that collect and process data to identify anomalies and increase heating equipment’ lifetime, as well as its efficiency. *PASMO* is an experimental platform for smart mobility applications and services, focusing on automotive mobility. These projects rely on hundreds of IoT sensors (in one of them, we used 750 physical devices) deployed on the customers’ locations, gathering data for several months, over a wide range of product manufacturers, networks, and scenarios. Even if not reaching the millions of devices aimed in these platforms, these already provided environments rich enough for assessing the limitations of SCoTv1. Both projects are described with more detail in Section 4.

Solutions such as cloud computing, containerisation, and container orchestration can eliminate most of the limitations associated with traditional deployments and can be used to develop flexible IoT platforms. Integrating the aforementioned software solutions potentially allows deploying IoT platforms quickly and with new and improved features. Thus, the main contribution of this work is to describe the deployment of the SCoT 2.0 platform, which is based on containers (instead of a single monolithic service as SCoTv1) and several innovations associated with scaling concerns. In particular, we describe in detail the modular data gateway, which can store any data into a persistent storage solution (see Section 3) and lessons learned when deploying large scale IoT scenarios.

This paper is organised as follows. Section 2 exposes previous and theoretical background information relevant to this work. Section 3 describes in detail the implementation of the architecture described in this paper. In Section 4, we describe the IoT scenarios that are currently being supported by the proposed platform. Finally, Section 5 presents our conclusions and future work that we aim to pursue.

## 2. State of the Art

**Figure 1:**
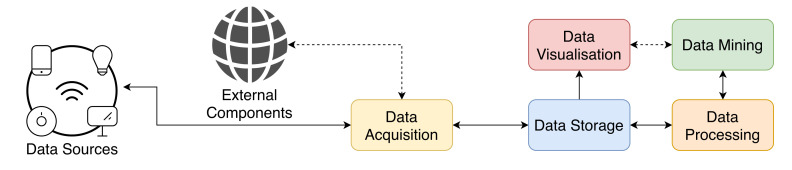
Key components that compose an IoT platform.

The construction of an effective and functional IoT platform requires some key macroscopic components [7], as depicted in Figure 1.

*Data Acquisition* is the component responsible for retrieving data that will be processed next. It achieves this by connecting sources with the remaining architecture, often providing authentication and authorisation mechanisms.

The following component, named *Data Storage*, is responsible for the persistence of all the necessary data acquired previously, and then the *Data Processing* component controls the transmission between storage and the upper components [8].

The *Data Mining component* shall process the data to identify important features and discover patterns based on them. The pipeline implementation is crucial to the platform since it may limit the system ability to scale correctly [9,10].

There are two main approaches to *Data Processing*, which we describe next with greater detail: Batch Processing and Real-Time (or Stream) Processing.

Finally, the *Data Visualisation* component shows valuable information [11], and it typically adapts to each scenario, the users, and purpose of the system.

In the following subsection, we detail the available solutions for each of the key blocks described previously and comment on some implementation aspects in different (often commercial) solutions.

### 2.1. Data Acquisition

The first key component is **Data Acquisition**, which is the component that connects the sensors and actuators to the platform, filling the gap between hardware and the processing frameworks (through remote and heterogeneous networks). Usually, it is implemented as a multi-protocol broker. The authors describe in detail the different data acquisition platforms [12,13]. The most relevant alternatives are described in Table 1.

Although Mosquitto is a highly used broker in IoT scenarios due it being fast and lightweight, it only supports the MQTT protocol. The other two brokers support several communication protocols, which enables the connection of heterogeneous devices.

### 2.2. Data Storage

The majority of IoT scenarios produce data that are better perceived as time series [14,15,16,17] and better processed using techniques developed for sequential series of points. Time Series Database (TSDB) has become a common storage solution for such scenarios.

A TSDB is optimised for storing a sequence of values (a pair composed by the time and the value). In this sense, time-series data can be defined as “*time series is a collection of temporal data objects*” [18]. In many cases, the time-series repositories will utilise compression algorithms to manage the data efficiently. Although it is possible to store time-series data in other types of databases, the design of these systems usually imposes unnatural relations that are not well supported within the internal model of the underlying system. This leads to a loss of performance or data repetition. Most importantly, TSDB have additional features, such as allowing processing data in alternative manners, sometimes including more advanced mathematical expressions. Those databases are built for dealing with data that change over time. Some of the most used are represented in Table 2.

Several authors have proposed the use of Blockchain technology as the means to develop storage solutions for IoT [19,20,21]. The authors defend that data storage based on Blockchain tend to be more secure than the remaining storage solution. However, these solutions require substantial computational power. As such, they do not tend to be used widely.

### 2.3. Data Processing

The processing pipeline is crucial for the execution of large scale scenarios since it tends to be the major bottleneck of the IoT platform. The processing pipeline can be deployed with two different strategies: Batch Processing and Real-Time (or Stream) Processing.

Batch Processing follows a strategy where data are processed as one large batch, creating the need to provide random access to all the individual pieces of data. There are several frameworks for batch processing [22]; in Table 3, we detail the most common ones.

Real-Time Processing, often called Stream Processing, requires the continuous availability of computing and network resources. There are two types of data streams: bounded and unbounded. Unbounded streams have a start but no defined end. They do not terminate and provide data as the data are generated. Bounded streams have a defined start and end. There are also several frameworks for stream processing; the most common ones are described in Table 4).

### 2.4. Data Visualisation

Data Visualisation [31] is a service that presents the acquired data in a graphical representation. Effective visualisation helps end-users to analyse and reason about data and related patterns. It makes complex data more accessible, understandable, and usable. When dealing with data that include thousands or millions of data points, automating the process of creating a visualisation, at least in part, allows a common user to better understand the underlying patterns in the data.

Processing, analysing, and communicating these data present ethical and analytical challenges for data visualisation [32]. Moreover, this block provides an accessible way to see and understand trends, outliers, and patterns in data. Considering some of the most used tools for Data Visualisation, several platforms are described in Table 5.

### 2.5. Containerisation

In this subsection, we discuss the usage of containerisation and microservices applied to IoT scenarios. Containers enable developers to define and build their software environments and then run them on top of various resources in a portable, reproducible way [33]. Microservices architecture allows the development of a distributed platform as a set of independent components that work together. This architecture is not novel, but when applied in cloud systems, it increases the platform scalability and reliability [34].

The authors of [35] proposed a modular and scalable architecture based on lightweight virtualisation for Industrial Internet of Things (IIoT) scenarios. In the proposed architecture, each component has embedded docker, applications (divided in small services), and implemented inside containers. By adopting the proposed architecture, reliability can be achieved through the application of orchestration rules that can ensure service recovery in case of a failure, and system resilience can be improved by including redundancy at different layers.

In [36], the authors state that given the nature of IoT scenarios, the inherent applications should be distributed, secure, and support heterogeneity. They propose an IoT platform based on the microservice models. This platform is leveraged on a SAVI cloud, a two-layer academic cloud, including a core in Toronto and seven smart edges across Canada.

### 2.6. IoT Platforms

Before detailing our SCoT2.0 IoT platform, it is relevant to analyse existing solutions and services. As previously stated, this platform is an evolution over previous SCotv1 [4,5], and as such, most of the background analysis is already presented on them. Nevertheless, this section briefly summarises the key lines described in previous works.

Amazon Web Services (AWS) IoT [37,38] are a set of cloud services available through AWS that connect IoT devices to other devices and/or AWS cloud services. AWS IoT provides device software that can help customers integrate IoT devices into AWS IoT-based solutions. The AWS private cloud provides on-demand cloud computing platforms and APIs to individuals on a metered pay-as-you-go basis.

ThingWorx [39,40] is an IoT platform that provides the necessary services to bind the automation, optimisation, control, and monitoring into a single framework. The platform provides the tools to map our application design into a working model. The platform is a collection of modules that deliver the flexibility, capability, and agility establishment required to implement IoT applications.

ThingSpeak [41] is an open data platform for IoT that enables the development of IoT applications. It provides a middle-layer that integrates the acquired data with a variety of third-party platforms, systems, and technologies, including other leading IoT platforms, such as ioBridge, Arduino, and the numerical computing software MATLAB from MathWorks. This allows the customers to have access to a well-known third-party for the tasks of analysing and visualising the acquired data with ease.

Google Cloud IoT [42] allows the customers to develop and deploy their IoT applications on the services provided by the Google Cloud Platform. It runs on the same infrastructure that Google uses internally for its end-user products. This enables the customers to only worry about the IoT application and rely on the Google services for scalability, reliability, and even for advanced machine learning capabilities and an integrated software stack that allows predictive maintenance scenarios.

Microsoft Azure IoT [43] follows a similar strategy to Google Cloud IoT; it also offers a cloud computing service that relies on the cloud services provided by Microsoft. One of the main differences is the possibility of using preconfigured solutions.

The Bosch IoT Suite [44] is a set of cloud services and software packages for the development of IoT applications. It is available as Platform as a Service (PaaS) for customers, who can quickly build and implement cloud-based and highly scalable IoT applications.

The previously mentioned platforms are some of the most used to build IoT applications. These platforms have two main advantages: I) the platforms rely on the parent company cloud infrastructure (leading to increase scalability and reliability), and II) the customers can connect to third-party services (usually part of the parent company) to access several advanced features, such as persistent storage, data analytics, and data visualisation. Several authors have shown the positive impact of cloud-based solutions [45,46] as the basis of an IoT platform.

However, these platforms offer the bare-bones for IoT devices (through a multi-protocol broker) and some type of persistence. Since its conception, the SCoT platform intends to go a little further. Our proposal for an IoT platform also has a focus on scalability and reliability but provides data representation agnostic storage (the usage of smart parsers) and easy integration with data mining and visualisation services (without relying on a parent company’s external services).

SCoT is a platform developed to deal with massive IoT scenarios. It is an evolution over our previous solution, named SCoTv1 [3,4,5], that aimed to develop a generic platform for the integration of IoT scenarios in telecommunication environments. Therefore, it implements functions related to device management, service integration, and applications under a telco service provision model.

After long experience with this previous implementation, and drawing from the experience of supporting different projects, sensor types, and scenarios, it was possible to identify the following limitations:

- Monolithic architecture—there was no redundancy available, or the redundancy was limited by the inherent construction centred in service layers with common service functions.
- Difficult to manage—it is challenging to add new services or to debug issues. In particular, scalability and performance issues.
- Under-performing in unexpected high traffic scenarios—infrastructure might not be able to handle a high number of connections or data objects being passed. Objects were duplicated and processed by a large number of service functions, and an error could propagate to further layers without being noticed.
- Difficult to redeploy the same platform—the services were deployed mostly manually and do not allow redeploying the same infrastructure quickly and automatically, which was already cumbersome for telecom operations but impossible to use in other IT operation scenarios.
- Possible conflicts between services in the same machine—One malfunctioning service can affect other services running in the same machine (e.g., a memory leak, data corruption).

Although these issues do not question the basic design paradigm for the IoT platform, cumulatively, they bring major roadblocks for the widespread usage of IoT. This work aims to smooth these roadblocks by developing an improved IoT platform and demonstrating its usefulness.

## 3. Scot Architecture and Implementation

In this section, we describe the relevant implementation architectural details regarding the proposed improved IoT platform (SCot2.0) and present some lessons learned from devising, building, and deploying these types of platforms.

As stated in the previous section, the redesign of the SCoT platform should optimise resource usage and provide an adaptable platform capable of dealing with massive and dynamic IoT scenarios. The select way to optimise resource usage was through a careful division of the monolithic platform into microservices that can be easily deployed into individual containers. The proposed solution tries not only to eliminate these limitations but also to bring new benefits such as:

- Distributed architecture—Implemented using a multi-node distributed platform, which allows the distribution of the workload amongst the available workers and making it fault-tolerant by keeping track of the nodes’ health;
- Service replication with load balancing—The proposed implementation allows the replication of any service to improve scalability. Furthermore, the incoming traffic is distributed amongst all replicates to maximise the performance;
- Easier to scale the infrastructure and/or applications—This implementation facilitates the scaling operations of either the cluster or the services of an application, allowing a near-zero downtime;
- Excessive work in updating the platform—The fully custom implementation brought large challenges to code base maintenance as types of software evolve, and multiple security bugs become known. Keeping the whole platform updated requires meaningful proprietary effort.

**Figure 2:**
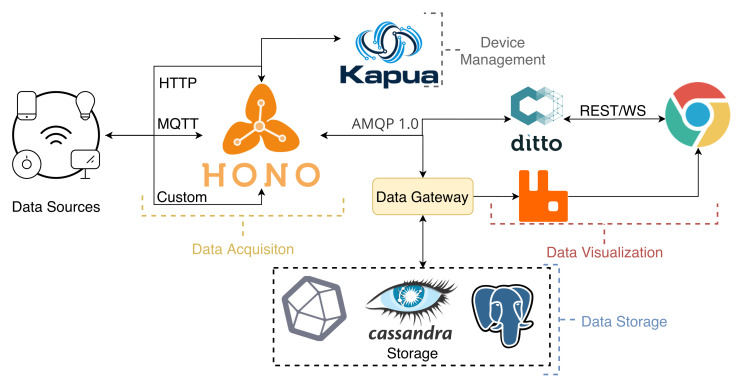
Redesign of the SCoT’s architecture to support dynamic IoT scenarios.

The SCoTv1 platform was redesigned in terms of software implementation and some of its modules, as depicted in Figure 2. SCoTv2 is now based on three key services from the Eclipse Stack (Hono, Ditto, and Kapua), further extending with custom services. These components were chosen since they cover most of the initial requirements, and they provide a high level of integration between them. They are part of the Eclipse IoT (https://iot.eclipse.org/ accessed on 1 October 2021) initiative that is well known and heavily used in this area. This approach allows the system to be much easier to update while maintaining essential performance functions fully under the control of the platform owners. The platform is deployed within a Docker Swarm consisting of three manager nodes and five worker nodes. The three manager nodes allow one to fail without bringing the platform down. Only the persistence services are maintained outside of the swarm, the persistence databases, and an NFS server for shared configurations. This allows the swarm to move the services as it sees fit since each server is stateless. Stateless, in this context, means that the data persistence is managed externally.

It is also important to mention that both the broker (Hono) and the digital twin service (Ditto) have user credentials and access policies. Each sensor/gateway has its own access credentials, and the users that have access to them can also be controlled using different policies.

Eclipse Hono (https://www.eclipse.org/hono/ accessed on 1 October 2021) is an IoT message broker that provides remote service interfaces for connecting large numbers of IoT devices to a back end and uniformly interacting with them regardless of the communication protocol. Hono collects telemetry data from these devices through Advanced Message Queuing Protocol (AMQP), MQTT, Constrained Application Protocol (CoAP), or Hypertext Transfer Protocol (HTTP) and discloses it through an API to an AMQP network, which is inherently distributed. It supports two data paths for events and telemetry, plus a control path. We deployed the suite based on containers in an infrastructure providing support for a wide range of orchestration environments.

Eclipse Ditto (https://www.eclipse.org/ditto/ accessed on 1 October 2021) implements a software pattern called “digital twins”. A digital twin is a digital representation of a physical “Thing”, e.g., sensors and actuators. One advantage is the simplification of IoT applications since it decouples the application from the physical IoT device. Similar to the Hono framework, the Ditto framework can be deployed with a range of containers orchestration environments.

The connection between the Eclipse Ditto and Hono is not trivial. There are two types of connections that must be developed in order to have access to the data (incoming connection) and be able to send commands (outgoing connection) to the physical device. The incoming connection requires a mapping function that converts the physical device raw data into the JSON format supported internally by the Ditto component. The outgoing connection simply redirects the JSON sent to the digital twin to the physical device.

As stated in [47], there is an inherent complexity and heterogeneity of IoT devices; as such, defining incoming mappings for each physical device is quite taxing and requires human intervention. We developed two distinct approaches.

Initially, we developed a service that automatically created an incoming mapping for each new device based on a template database. However, we discovered that this approach has several disadvantages, as the number of active connections overloads the Ditto capability. Furthermore, whenever a device publishes any new type of information, it is necessary to add a new template into the database.

As an alternative to the previous approach, we then developed an automatic mapping function that analyses the JSON raw data, flattens it, and automatically generates the JSON message that is supported by the Ditto component (see Listing 1). The automatic incoming mapping is derived from the work presented here [48], where semi-structured formats are parsed with an EAV (entity-attribute-value) model. Another advantage is that this mapping function can be defined at the tenant level and not for each device individually, decreasing the number of active connections. This alternative has been shown to be scalable, and our implementation is currently supporting two large IoT scenarios (see Section 4).

**Listing 1: Pseudocode extract from the ditto incoming mapping**
**function** mapToDittoProtocolMsg(headers, textPayload,
bytePayload, contentType) {
  **let** rv = {};
  **try** {
    **let** jsonData;
    **if** (contentType == “application/json”) {
      jsonData = JSON.parse(textPayload);
    } **else** {
      **let** payload = Ditto.asByteBuffer(bytePayload);
      jsonData = JSON.parse(payload.toUTF8());
    }
    **let** stack = [[jsonData, “”]];
    **while** (stack.length) {
      **let** tmp = stack.pop(), root = tmp[0], preffix = tmp[1];
      for (**let** key **in** root) {
        **let** value = root[key];
        **if** (!!value && value.constructor === Object) {
          **let** new_preffix;
          **if** (preffix) {
            new_preffix = preffix.concat(“.”, key);
          } **else** {
            new_preffix = key;
          }
          stack.push([value, new_preffix]);
        } **else** {
          **let** new_preffix;
          **if** (preffix) {
            new_preffix = preffix.concat(“.”, key);
          } **else** {
            new_preffix = key;
          }
          rv[new_preffix] = {“properties”:{“value”:value}};
        }
      }
    }
  } **catch** (e) {
    **let** byteBuf = Ditto.asByteBuffer(bytePayload);
    rv = {“raw”:{“properties”:{“value”:byteBuf.toBase64()}}}
  }
  **return** Ditto.buildDittoProtocolMsg(“{}”, headers[“device_id”],
  “things”, “twin”, “commands”, “modify”, “/features”, headers, rv);
  }

Furthermore, we also instantiated a RabbitMQ cluster to provide real-time access to the data stream, besides the access also provided at the level of the Digital Twin.

Eclipse Kapua (https://www.eclipse.org/kapua/ accessed on 1 October 2021) is used to manage and integrate devices and their data to provide a solid foundation for IoT services for any IoT application. In short, it is responsible for the device management, registration, and authentication.

The proposed platform also includes custom destined services. These include a *Data Gateway* that connects to several databases.

As stated in [47], IoT scenarios are inherently complex due to the data heterogeneity. It becomes rather difficult to have a single database model to store the data and provide a meaningful structure for *Data Processing* components. This is one of the focuses of our platform, we developed a *Data Gateway* that uses a smart parser to transform the data and store it correctly in the instantiated databases. Three different databases (with replicated data) are used in our current deployment: Apache Cassandra, PostgreSQL, and InfluxDB. Each one of them is a mature representation of NoSQL, Relational, and Time-Series databases, respectively. Depending on the scenario’s requirements, the customer can retrieve data from the appropriate database. This approach was selected since the storage’s space is relatively cheap, and the use of the adequate database facilitates data processing tasks and integration with additional services.

**Figure 3:**
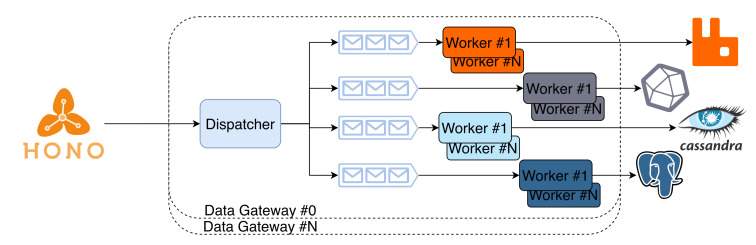
High-level architecture of the Data Gateway service.High-level architecture of the Data Gateway service.

The *Data Gateway* bridges the Hono framework with the *Data Storage* and the real-time broker that are themselves exposed to services. This service was designed to scale vertically and horizontally, as depicted in Figure 3. The Gateway is a parallel construct, with differentiated processing workers and a queue for each database backend. Each worker consumes messages from the broker and stores them into the respective database (horizontal scaling), applying transformations required to make the data compatible with the database. Furthermore, there can be more than one gateway instantiated, as well as more than one worker per database per gateway, achieving high levels of parallelism and scalability. The ingress portion of the gateway constitutes a consumer at the AMQP network used after Hono.

Since the Data Gateway is designed to just be a consumer in the AMQP network used after HONO, horizontal scaling is achieved by requesting additional replicas that can be hosted in any node. If *N* replicas are requested, then *N* AMQP consumers are created. Since every Data Gateway subscribes the same queues, the messages are load-balanced automatically. Vertical scaling is achieved by increasing the number of workers (processes) on each replica that connects and stores data on each data storage system. It begins by creating *D* different queues, one for each data storage system, and then instantiates *k* processes for each worker, passing them their respective data storage system queue to consume from.

The number of workers is always equal to D×k+1, where *D* is the number of underlying data storage systems and *k* is the configured number of workers per data storage system. The extra one process is the dispatcher that subscribes to the AMQP network and feeds messages to the worker queues.

The schema of the Relational and NoSQL databases was based on previous work [48], where the structure is derived from implicit data structures. The InfluxDB has a unique internal structure; we had to develop a recursive flat parser to implement a generic storing method. This parser is capable of flattening any JSON document (similar to the parser defined for the automatic mapping Listing 1) and guessing variable types, names, and other metadata from the names used by sensors. The flattened JSON is then parsed into individual components and inserted into the InfluxDB as individual streams. As long as the properties have meaningful names, direct categorisation is possible. In other cases, properties of the data series can expose the real semantics and allow automatic categorisation.

Finally, as previously stated (in Section 2), the *Data Mining component* is a key component in the platform. However, in previous work, we already detailed the most important details regarding their components [6]. The inner workings of this component is out of the scope of this work.

## 4. IoT Scenarios

In this section, we describe with some detail the two IoT projects that are currently being supported by the proposed platform and have been used to evaluate its (horizontal) scalability. It is important to mention that the current deployment of the platform was deployed using Docker Swarm containerisation.

The first, named Smart Green Homes (SGH) (https://www.ua.pt/pt/smartgreenhomes/ accessed on 1 October 2021), is a joint cooperation between the University of Aveiro and Bosch with the objective of developing smart solutions for thermotechnology. One of the areas of this project is identifying the comfort temperature of the residents. To achieve this, several volunteers fitted their houses’ with multiple environmental and presence sensors. All previously mentioned sensors are publishing data into the proposed platform.

The second project is named PASMO [49,50,51]; the main objective is the design, field implementation, and provision of a platform for intelligent mobility, which is open to participative experimentation of companies that can collaborate to test (technology) and validate (market) equipment, protocols, processes, applications, standards, and services. One scenario explored by this project is the development of Intelligent Transportation Systems (ITS) where the traffic conditions and the weather are monitored continuously. Furthermore, notifications related to the traffic conditions are sent to users of the platform. To this end, multiple traffic and environmental sensors are deployed alongside relevant road junctions in the region of Aveiro.

Currently, there are more than 750 physical devices registered within the proposed platform. The large majority are the sensors associated with the previously mentioned projects, meaning that they are highly active devices that publish periodically. These projects generate on average 15 messages per second, comprising more than 50 GB of uncompressed data points. It is important to mention that the number of messages per second varies greatly during the day. The PASMO project monitors traffic conditions, meaning that at the beginning and the end of the day, there is a considerable burst of messages.

The Data Acquisition, Data Gateway, and Data Visualisation services all reside within a Docker Swarm consisting of three manager nodes and five worker nodes. The three manager nodes allow one to fail without bringing the entire swarm down. The manager nodes have 4 CPUs, 8 GB of RAM, and 32 GB of storage each, **while** the worker nodes have 4 CPUs, 16 GB of RAM, and 40 GB of storage each.

In terms of storage, the NFS used in the platform to provide persistence has 32 GB available with 1.56 GB currently in use. The Postgres and InfluxDB data stores have both 8 CPUs, 16 GB of RAM, and 160 GB of storage. While InfluxDB is using only 3.20 GB of storage, Postgres is using 48.70 GB. Cassandra is running in a four-node cluster, and each node has 4 CPUs, 16 GB of RAM, and 100 GB of storage. All four nodes use 46.88 GB of disk space due to a replication factor of three, allowing a node to fail without losing any of the data. It is worth noting that while the memory load of every node discussed so far is in the 2% to 7% range, the cassandra nodes use around 32%. Thus, InfluxDB uses the least amount of resources, while comparatively, Postgres needs a lot more storage and Cassandra a lot more memory. A summary of the loads of each machine is depicted in Table 6.

Once the platform was deployed with a stable release of the Data Gateway, the only downtime was caused by the misconfiguration of one or more components. This occurred three times in the span of a year, and the major cause was mainly related to logging not being properly rotated, which led to disk space issues. However, one of the downtime incidents was related to expired certificates, which led to the Hono components being unable to communicate with one another.

The platform is being used by two large-scale IoT projects; this heavily limits the evaluation we can perform on it without interfering in the projects. Nevertheless, to demonstrate the capabilities of the current deployment, we place three probes within the platform: bridge, ditto, and sensor. The latency values were measured simultaneously with the previously mentioned IoT projects running. The probe within the bridge measures the time that a message from a sensor takes to reach the bridge. The communication path for this probe is MQTT → AMQP. Similarly, the probe in Ditto measures the time a message from a sensor takes to reach the digital twin. The communication path for this probe is MQTT → AMQP → WebSockets (WS). Finally, the last probe measures the time that a command message issue in the digital twin takes to reach the physical sensor. The communication path for this probe is HTTP → AMQP → MQTT. The results are presented in Table 7 and give an idea of the expected performance for the platform.

We also developed a virtual sensor that sends messages at a fixed rate of 100 messages per second. Four different configurations were deployed with one, two, four, and eight virtual sensor. Each sensor sent 10 K messages, and the previously mentioned probes store the latency of the messages. We only used two probes: bridge and ditto. The communication path is similar to the one described previously. We did not use the sensor probe since the control channel was being heavily used by the IoT projects.

**Figure 4:**
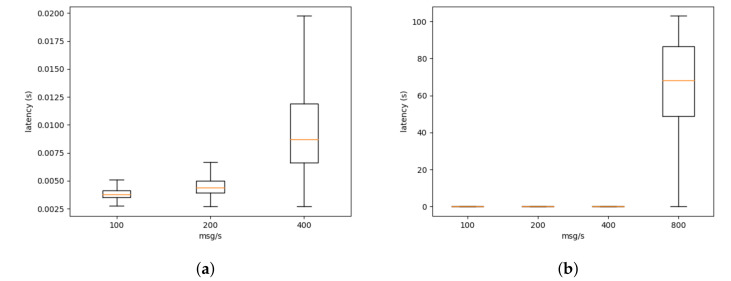
Latency at the bridge level. Due to the scaling issues (**a**) only shows from 100 to 400 msg/s while (**b**) shows from 100 to 800 msg/s.

In Figure 4, we can see the latency at the bridge level. The latency increases linearly with the number of messages (the number of messages is growing exponentially) when the sensors send less than 800 messages per second. However, when the virtual sensors are sending 800 messages per second (see Figure 4b), the platform reaches its maximum capacity given the current deploy.

**Figure 5:**
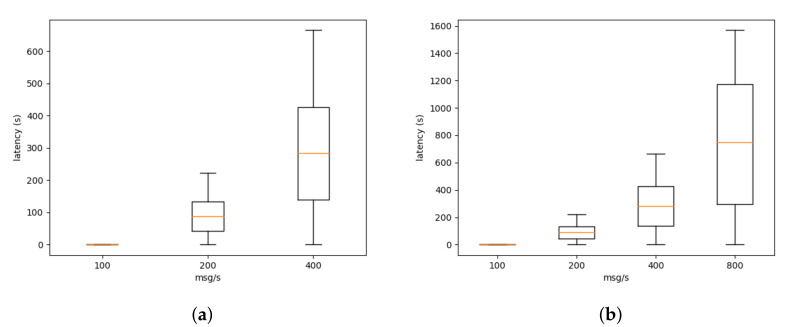
Latency at the ditto level. Due to the scaling issues (**a**) only shows from 100 to 400 msg/s while (**b**) shows from 100 to 800 msg/s.

Similarly, in Figure 5, we can see the latency at the ditto level. The latency at the ditto level is considerably greater than at the bridge level. This is explained by the execution of a mapping function (see Listing 1) within the connection between ditto and the bridge. In our current deployment, there is a single mapping function per tenant. As stated previously, this limits the number of open connection in the ditto component, increasing the stability of the service. However, for this test, a single mapping function (and connection) is being used to process all the messages from the sensors. This increases the latency greatly.

As previously stated, these experiments were performed on the platform, while it was being used by the IoT projects. This limits the amount of perturbation we can inject into the platform to extract performance metrics. Nevertheless, our evaluation shows that the platform in its current state can support less than 800 messages at the bridge level and less than 200 messages at the ditto level. That performance level is sufficient for the currently deployed project. As discussed in Section 3, given the implementation of the platform as microservices, it is possible to scale the platform whenever higher levels of performance are necessary.

## 5. Conclusions

The establishment of cloud computing, allied with the evolution of containerisation, orchestration solutions, and the complementary Software Configuration Management tools, has changed the way software solutions are projected and deployed. Having this set of technologies in mind, it is possible to build robust supporting platforms that offer excellent performance and new features, such as fault tolerance, load balancing, and easy infrastructure replication for the most different scenarios.

This paper describes the latest iteration of our IoT platform devised as a robust software architecture able to handle large-scale data acquisition, processing, and visualisation, targeting Big Data scenarios. The platform was developed based on independent services that can be deployed as containers in a private cloud. The solution allows customers to integrate several sources and obtain relevant information only by connecting their sensors. Some aspects were evaluated to verify its effectiveness, such as the design, storage capacity, and scalability. The real scenarios deployment has proven that the system is scalable and able to process the necessary amount of data, even while relying upon many open-source components.

The platform is being actively used by two large-scale IoT projects. It was dimensioned to support the performance requirements from the projects. Regardless, some limitations will be addressed in future work. First, the platform was deployed using docker swarm since one initial evaluation has shown that it was faster at deploying and recovering from errors than Kubernetes. However, the docker swarm mode is no longer being maintained (https://www.mirantis.com/blog/mirantis-acquires-docker-enterprise-platform-business/ accessed on 1 October 2021). We already started to research how to properly migrate our platform from docker swarm to Kubernetes while taking advantage of some advanced features from the latter. Second, the Hono and Ditto versions are considerable outdated at this point. To maintain the platform (due to the use cases we are supporting) and keep the current APIs stable, we are still using outdated versions of these services. The next iteration of the platform will update these services and adjust the custom component to have a seamless integration and allow for continuous software updates. Finally, the ML is becoming an integral part of most IoT projects, and, as such, we intend to include more services dedicated to this area.

## Figures and Tables

**Table 1:** IoT Messages brokers review.

Broker	Advantages	Disadvantages
Mosquitto	Message Queuing Telemetry Transport (MQTT) Message Broker; Lightweight	Protocol limitation
RabbitMQ	Multi-protocol broker; Easy to scale	Limited features for IoT
Hono	Multi-protocol broker; Queues with different semantic	Computationally heavy

**Table 2:** Time-series databases review.

TS Database	Advantages	Disadvantages
InfluxDB	Fast storage; Time-series data retrieval	Limited scalability
TimescaleDB	SQL scalability; Easy interpretation	Large time-based queries
OpenTSDB	Scalability; Storage as a server	Hadoop knowledge
Redis	Wide variety of data types; Open source	No joins operations or conventional queries
MongoDB	BSON document as a unit; Horizontal scalability	Non-indexed queries; Hardware requirements
Apache Cassandra	Fault-tolerant; Linear scalability	No unanticipated queries
MapR Database	High-performance; Analytic capabilities	Non-indexed queries

**Table 3:** Batch Processing frameworks review.

Framework	Main Characteristics	Useful Scenarios
Hadoop MapReduce [23]	Independent clusters	Large amounts of data
Hadoop HDFS [24]	Intermediate results	Shared repository of data
Google Dremel [25]	Reduces the CPU overhead	Multiple features

**Table 4:** Stream Processing frameworks review.

Framework	Main Characteristics	Useful Scenarios
Apache Spark [26]	Supports Lambda architecture	Applications with diverse data sources
Apache Storm [27]	Unbounded streams of data	Real time analytics
Apache Flink [28]	Unbounded and bounded data	Fault-tolerant applications
Apache Samza [29]	Multiple streams	Fault tolerance and buffering
Apache Druid [30]	Real-time analytics on large datasets	Clusters with several nodes

**Table 5:** Time-series databases review.

TS Database	Advantages	Disadvantages
Grafana	Several time-series data storage; Allow notifications and alerts	Full-text data querying not permitted
Kibana	Data querying and analysis; Several data representations	Work only with Elasticsearch
Graphite	Highly scalable; Render on demand	Specific database
Prometheus	Multi-dimensional data model; Flexible query language	Not viable for anomaly detection

**Table 6:** Specifications and loads of the servers used to deploy our platform.

	CPU		Memory		Disk	
	**Cores**	**Load (%)**	**Total (GB)**	**Load (GB)**	**Total (GB)**	**Load (GB)**
manager-0	4	1.86	8	7.03	32	3.57
manager-1	4	1.67	8	6.04	32	2.68
manager-2	4	3.36	8	6.86	32	4.35
worker-0	4	1.87	16	3.79	40	3.50
worker-1	4	1.70	16	3.03	40	1.43
worker-2	4	1.60	16	3.72	40	3.82
worker-3	4	2.28	16	6.94	40	3.33
worker-4	4	1.65	16	4.07	40	8.52
nfs-server	4	1.42	8	2.98	32	1.56
postgres	8	0.71	16	1.99	160	48.70
influxdb	8	1.28	16	4.12	160	3.20
cassandra-0	4	1.63	16	31.85	100	9.50
cassandra-1	4	1.94	16	32.29	100	13.16
cassandra-2	4	1.95	16	31.49	100	10.98
cassandra-3	4	2.18	16	32.07	100	13.24

**Table 7:** Latency of different probes placed within the proposed platform.

Probe	Latency (Seconds)
Bridge	0.05±0.02
Ditto	0.05±0.02
Sensor	0.15±0.01

## Data Availability

Not applicable.
